# Everolimus-Induced Pneumonitis in Patients with Neuroendocrine Neoplasms: Real-World Study on Risk Factors and Outcomes

**DOI:** 10.1093/oncolo/oyab024

**Published:** 2022-02-01

**Authors:** Rodrigo G Taboada, Rachel P Riechelmann, Carine Mauro, Milton Barros, Richard A Hubner, Mairéad G McNamara, Angela Lamarca, Juan W Valle

**Affiliations:** 1 Department of Medical Oncology, A.C.Camargo Cancer Center, São Paulo, Brazil; 2 Department of Medical Oncology, The Christie NHS Foundation Trust/Division of Cancer Sciences, University of Manchester, Manchester, UK; 3 Division of Cancer Sciences, University of Manchester/The Christie NHS Foundation Trust, Manchester, UK

**Keywords:** neuroendocrine tumors, everolimus, pneumonitis

## Abstract

**Background:**

Everolimus-induced pneumonitis (EiP) has been poorly studied in patients with neuroendocrine neoplasms (NEN) outside clinical trials. The aim of this study was to evaluate the incidence, risk factors, and outcomes of EiP in patients with NENs using real-world data.

**Methods:**

Retrospective study of everolimus-treated patients with advanced NENs. Imaging reports were systematically reviewed for the presence of pneumonitis. Clinical features and treatment profiles for EiP were summarized. Overall survival (OS) was calculated from the initiation of everolimus to the date of death or last follow-up using the Kaplan-Meier method.

**Results:**

A total of 122 patients were included. Median age at start of everolimus was 62 (19-86) years, 62% (76/122) were male, and half were from pancreatic origin (62, 51%). Twenty-eight patients (23%) developed EiP: 82% grade (G)1 or G2, 14% G3 and 4% G4. The median time to EiP was 3.6 (0.8-51) months. Primary tumor site, concurrent lung disease, smoking history, and prior therapies were not associated with the onset of EiP. Patients who developed EiP had longer time on everolimus treatment (median 18 months vs 6 months; *P* = .0018) and OS (77 months vs 52 months; *P* = .093). Everolimus-induced pneumonitis was a predictor of improved OS by multivariable analysis (HR 0.39, 95% CI 0.19-0.82; *P* = .013).

**Conclusion:**

Everolimus-induced pneumonitis in the real-world clinical setting is present in one quarter of patients with NENs receiving everolimus and often occurs early. While risk factors for EiP were not identified, patients with EiP had improved survival.

## Teaser

Everolimus-induced pneumonitis has been poorly studied in patients with neuroendocrine neoplasms. This article evaluates the incidence, risk factors, and outcomes of everolimus-induced pneumonitis in patients with neuroendocrine neoplasms using real-world data.

**Implications for Practice**

Neuroendocrine tumors have their incidence steadily rising and everolimus is a commonly used treatment. Everolimus-induced pneumonitis (EiP) is a frequent adverse event, occurring in nearly one quarter of patients. Our study demonstrated for the first time the association between the occurrence of EiP and an improvement in survival in patients with neuroendocrine tumor. This intriguing finding, if confirmed, could have clinical implications to reinforce adequate management of this adverse event, with the goal of preserving everolimus therapy when possible and thus offering the best care for patients with neuroendocrine tumors.

## Introduction

Neuroendocrine neoplasms (NEN) are a heterogeneous group of cancers originating from neuroendocrine cells diffusely present in the body, with variable prognosis and response to standard therapies. The median survival of patients with NENs varies according to primary site, degree of differentiation, grade, and the occurrence of distant disease.^1^ However, other factors, such as treatment-related adverse events, also influence outcomes. Everolimus is an oral inhibitor of the mammalian target of rapamycin (mTOR) with anti-cancer activity in multiple tumor types, including renal cell carcinoma (RCC), mantle cell lymphoma, breast cancer, and well-differentiated NENs. Everolimus is a targeted agent with anti-tumor activity across a broad range of well-differentiated NENs, hereafter neuroendocrine tumors (NET), from distinct origins. As compared with placebo, everolimus significantly prolonged median progression-free survival (PFS) among patients with progressive advanced pancreatic NET,^2^, nonpancreatic/nonfunctional NET^3^ and, by investigator assessment (but not by central evaluation), in combination with octreotide in advanced functioning NET.^4^

The known side-effect profile of everolimus includes pneumonitis. This adverse event consists of a spectrum that can range from asymptomatic patients with radiological evidence of lung inflammation with interstitial infiltration, and opacities, to symptomatic patients who require hospitalization, directed therapy, and everolimus discontinuation. In clinical trials of everolimus in NET, the reported rates of pneumonitis of all grades ranged between 12% and 17%.^2-4^ Pivotal studies of everolimus in breast cancer and RCC reported similar rates of all grades pneumonitis, ranging from 8% to 16%.^5-8^ A pooled analysis of trials with everolimus used in different tumor types demonstrated that 2.4% of patients developed grades 3-4 pneumonitis.^9^ A multicenter retrospective study in patients with gastro-entero-pancreatic and lung NETs, however, showed a 3 times higher incidence of everolimus-associated pneumonitis.^10^ Among 169 patients with NETs, 8.3% experienced severe, grades (G)3-4, pneumonitis. Patients previously treated with peptide receptor radionuclide therapy (PRRT) and chemotherapy had a 12-fold increased risk for severe toxicity, without increasing the rate of G3-4 pneumonitis (7.9%). In this context of real-world evidence, a previous multicenter study by our group found that 21.6% of patients with a NET diagnosis on everolimus had G3-4 infections and 3.6% died.^11^

Limited data exist on the detailed characteristics and management practices for everolimus-induced pneumonitis (EiP) in patients with a NET diagnosis outside of clinical trials, particularly those of grade 1 or 2. The aim of the present study was to assess the incidence of clinical and subclinical pneumonitis in patients with NETs treated with everolimus, as well as factors associated with onset, management, and clinical outcomes in 2 comprehensive cancer centers, with experience in the multidisciplinary management of patients with NET.

## Materials and Methods

### Patients

This was a multicenter and multinational retrospective cohort study of eligible patients treated at the A.C.Camargo Cancer Center (São Paulo, Brazil) and The Christie NHS Foundation Trust (Manchester, UK), between January 2009 and September 2019. Hospital chart coding and electronic patient records were used to identify eligible patients and collect data.

Eligible patients had confirmed histological/cytological diagnosis of advanced/metastatic NET, received at least one dose of everolimus, and had a baseline and at least one more thoracic computed tomography (CT) imaging performed during everolimus treatment. Patients attending for a second opinion only who had their treatment elsewhere were excluded.

### Study Evaluation

Data captured included demographics (NET center, age at beginning of everolimus treatment, gender); clinical data (performance status at the beginning of everolimus defined as Eastern Cooperative Oncology Group, presence of functional syndrome, smoking history, history of chronic obstructive pulmonary disease [COPD], or prior chronic lung disease); pathology (differentiation of the primary tumor, history of lung metastases); treatment (line of therapy in which everolimus was used, prior treatments, need for treatment dose-reductions and/or interruptions, if combined with a somatostatin analogue, reasons for discontinuation).

The diagnosis of pneumonitis was made by establishing a correlation between radiologic reports and clinical judgment, as determined by the physicians caring for the patients. Therefore, the presence of pneumonitis was determined on review of the documentation in patients’ medical charts. If pneumonitis occurred, information on clinical features (grading using the Common Terminology Criteria for Adverse Events [CTCAE] version 4.0,^12^ respiratory symptoms, type of pulmonary radiology findings, investigational tests and therapies instituted for the management of pneumonitis), occurrence of concurrent everolimus-related adverse events if grades 3-4 by CTCAE, as well as clinical outcomes and follow-up were collected. Briefly, pneumonitis grades 1 to 4 corresponded to asymptomatic patients, symptomatic requiring medical interventions, oxygen indication, and life- threatening respiratory compromise, respectively. Grading assignment was based on patient’s symptoms, their severity, and the necessity of interventions related to the presence of respiratory symptoms, if any, as described in medical records. All images of cases suggestive of radiographic pneumonitis were reviewed for confirmation, by a Medical Oncologist (R.G.T.).

### Statistical Analysis

The primary endpoint was the frequency of pneumonitis development in patients with NETs receiving everolimus. Secondary objectives included prevalence of clinical (grade 2 or higher) and sub-clinical (grade 1) pneumonitis, impact of EiP on overall survival and identification of factors associated with EiP. The Fisher exact test for categorical data and *t*-test or Mann-Whitney test for continuous data, as appropriate, were used to compare demographics and clinical characteristics of patients with and without EiP. Normality distribution was assessed with Shapiro-Wilk test. A logistic regression analysis was performed to evaluate the association of pneumonitis of any grade with demographic or clinical factors. The follow-up time was calculated with the reverse Kaplan-Meier method.

Overall survival (OS) was calculated from the initiation of everolimus to the date of death or last follow-up and was estimated with the Kaplan-Meier method. Comparisons were performed using the log-rank test. Univariate Cox regression was used to assess the impact of covariates such as age at start of everolimus, line of treatment (first or second vs other), presence of lung disease, COPD, smoking history, the NET grade, the primary origin (pancreatic vs nonpancreatic), presence of lung metastases, rash, and development of EiP on survival analysis (OS); variables with a *P*-value of <.1 on univariate analysis were introduced in the Cox multivariable model. Patients who died during the first 3 months of treatment with everolimus may have had a lower chance of experiencing EiP (time dependent bias^13^). A landmark analysis was used to rule-out this by repeating the multivariable analysis excluding patients with early death (before 3 months). Time-to-event analyses used hazard ratios (HRs) with 95% confidence intervals (CI). Two-tailed *P*-values of <.05 were considered statistically significant. The end of follow-up was September 1, 2019. All statistical analyses were performed using Stata IC/16.0 software (StataCorp, College Station, TX).

### Ethical Considerations

The study was conducted in accordance with the protocol International Conference on Harmonization Good Clinical Practice guidelines and applicable laws and regulatory requirements. The protocol was approved by the Research Ethical Committee (A.C.Camargo) and by The Christie NHS Foundation Trust Audit Committee.

## Results

### Patient Characteristics and Everolimus Information

Overall, 122 patients were eligible and their characteristics are summarized in Table 1. The median age at start of everolimus was 62 (19-86) years and 62% were male. Half of the patients had a primary pancreatic origin (62, 51%). There was no statistical difference in age, gender, primary site, concurrent lung disease, smoking history, and prior therapies between patients with or without pneumonitis (Table 1).

**Table 1.** Patient and tumor characteristics.

		Pneumonitis		Total	*P-*value^a^
		No *N* = 94 (77%)	Yes *N* = 28 (23%)	*N* = 122	
Gender, male; *n* (%)		55 (59)	21 (75)	76 (62)	.11
Age	Median	60	65	62	.16
	Range	19-86	27-80	19-86	
ECOG PS, *n* (%)	0	26 (28)	10 (36)	36 (30)	.67
	1	59 (63)	15 (54)	74 (61)	
	2	9 (10)	3 (11)	12 (10)	
Comorbidities, yes; *n* (%)		67 (71)	22 (79)	89 (73)	.45
Lung disease; *n* (%)		13 (14)	4 (14)	17 (14)	.95
COPD, *n* (%)		2 (2)	1 (4)	3 (2)	.66
Smoker/prior smoker, *n* (%)		29 (35)	6 (25)	35 (33)	.34
Histological grade	1	22 (23)	6 (23)	28 (23)	.47
	2	67 (71)	20 (77)	87 (73)	
	3	5 (5)	0 (0)	5 (4)	
Primary, *n* (%)	Pancreas	44 (47)	18 (64)	62 (51)	.28
	Small bowel	15 (16)	5 (18)	20 (16)	
	Lung	20 (21)	3 (11)	23 (19)	
	UKP	11 (12)	1 (4)	12 (10)	
	Colon	2 (2)	0 (0)	2 (2)	
	Rectum	1 (1)	0 (0)	1 (1)	
	Appendix	1 (1)	0 (0)	1 (1)	
	Gastric	0 (0)	1 (4)	1 (1)	
Lung metastases, *n* (%)		22 (23)	8 (29)	30 (25)	.58
Line of treatment, *n* (%)	1	28 (30)	14 (50)	42 (34)	.18
	2	37 (39)	5 (18)	42 (34)	
	3	20 (21)	5 (18)	25 (20)	
	4	6 (6)	3 (11)	9 (7)	
	5	3 (3)	1 (4)	4 (3)	
Concurrent SSA, *n* (%)		34 (36)	9 (32)	43 (35)	.69
Prior SSA, *n* (%)		47 (50)	11 (39)	58 (48)	.31
Prior surgery, *n* (%)		41 (44)	12 (43)	53 (43)	.94
Prior TKI, *n* (%)		7 (7)	4 (14)	11 (9)	.27
Prior embolization, *n* (%)		11 (12)	5 (18)	16 (13)	.39
Prior radiotherapy, *n* (%)		12 (13)	1 (4)	13(11)	.16
Prior PRRT, *n* (%)		7 (7)	0 (0)	7 (6)	.13
Rash, *n* (%)		32 (34)	14 (50)	46 (38)	.12

ECOG PS: Eastern Cooperative Oncological Group Performance Status; COPD: chronic obstructive pulmonary disease; UKP: unknown primary; SSA: somatostatin analog; TKI: tyrosine kinase inhibitor; PRRT: peptide receptor radionuclide therapy.

*P-*values were calculated with Fisher’s exact test.

Percentages may not total 100 because of rounding.

Everolimus was used in two thirds of the cases in first- or second-line settings; 42 cases (34%) in each line. The median time on everolimus was 6.9 (0.2-117) months. Ninety-three (76%) patients stopped treatment due to progression, 10 (8%) due to adverse events (AEs) other than pneumonitis, 7 (6%) due to pneumonitis, and 12 (10%) were still on everolimus at the time of data-cut-off.

### Clinical Presentation and Management of Pneumonitis

**Figure 1.**
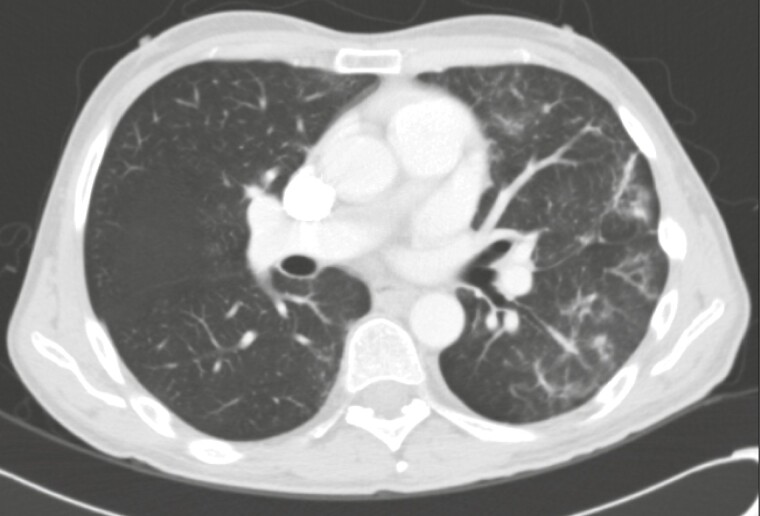
CT chest radiographic example of grade 1 pneumonitis. Unilateral multifocal subpleural ground-glass opacities.

The primary endpoint, frequency of any grade pneumonitis development in patients with NET receiving everolimus, occurred in 28 (22.9%, 95% CI 16-31%) patients. The median time to EiP was 3.6 (0.8-50.7) months. The most common radiological pattern was ground-glass opacities only, seen in 13 (46%) patients (Table 2). At diagnosis of pneumonitis, 9 (32%) had pneumonitis without respiratory symptoms (grade 1; Fig. 1). Six of those patients had an everolimus dose reduction, 4 had their treatment temporarily interrupted and, in all but one, their pneumonitis resolved (missing information for 1 patient). Fourteen (50%) had grade 2 pneumonitis, as determined by the physician caring for the patients, with the most common symptoms being cough and dyspnea. While 4 (14%) patients had to be admitted to hospital (grade 3); 1 patient had acute hypoxemic respiratory failure and was admitted to an intensive care unit, requiring oxygen therapy with Venturi mask. No interventions, such as bronchoscopy, were performed for evaluation of any of these cases. No pneumonitis-related deaths occurred.

**Table 2.** Radiologic abnormalities for patients with everolimus-induced pneumonitis.

Abnormality	No. of patients (*n* = 28)	%^a^
Ground-glass opacities only	13	46.4
Ground-glass and reticular opacities	4	14.2
Ground glass and consolidation	7	25
Ground-glass and reticular opacities and consolidation	4	14.2

Percentages may not total 100 because of rounding.

Dose reductions due to pneumonitis were needed in 32% (9/28) patients; while temporary and permanent discontinuation occurred in 50% (14/28) and 25% (7/28) of patients, respectively. All temporary discontinuation occurred in grades 1-2 pneumonitis (Table 3). Considering the patients who developed pneumonitis and had their treatment temporarily interrupted, 2 of them (2/14, 14%) had a recurrence of pneumonitis. The symptoms resolved in one and the other had minor sequelae at the follow-up cut-off. Overall, EiP resolved in 68% (19/28) of cases, 25% (7/28) recovered with minor sequelae, 1 patient with major sequelae, and unknown information for 1 patient.

**Table 3.** Everolimus-induced pneumonitis by CTCAE and management.

	Grade 1	Grade 2	Grade 3	Grade 4	All grades
Clinical grading, *n*	9	14	4	1	28
Everolimus therapy					
Dose reduction due to pneumonitis, *n*	6^a^	9	0	0	15
Temporarily interrupted for lung toxicity, *n*	4^a^	10	0	0	14
Permanent discontinuation for lung toxicity, *n*	0	2	4	1	7
Pneumonitis intervention					
Steroids initiated, *n*	0	7	4	1	12
Antibiotic p.o. initiated, *n*	2^a^	10	3	1	16
Antibiotic i.v. initiated, *n*	0	0	3	1	4
Oxygen supplementation, *n*	0	0	1	1	2
Pneumonitis outcome					
Resolved, *n*	8	9	1	1	19
Recovered with minor sequelae, *n*	0	4	3	0	7
Recovered with major sequelae, *n*	0	1	0	0	1
Died from pneumonitis, *n*	0	0	0	0	0
Unknown, *n*	1	0	0	0	1

Interventions done in patients with grade 1 pneumonitis were clinician’s decisions influenced by the images’ findings (asymptomatic patients).

CTCAE: Common Terminology Criteria for Adverse Events.

The most common grades 3-4 non-pneumonitis everolimus-related AEs were infectious complications (8 patients, 29%) followed by fatigue (5 patients, 18%) and mucositis (5 patients, 18%). Rash was detected numerically more frequently in the pneumonitis group (50%, 14/28) than in nonpneumonitis (34%, 32/94) cohort, but this was not statistically significant (*P* = .12).

### Pneumonitis and Survival Outcomes

**Figure 2.**
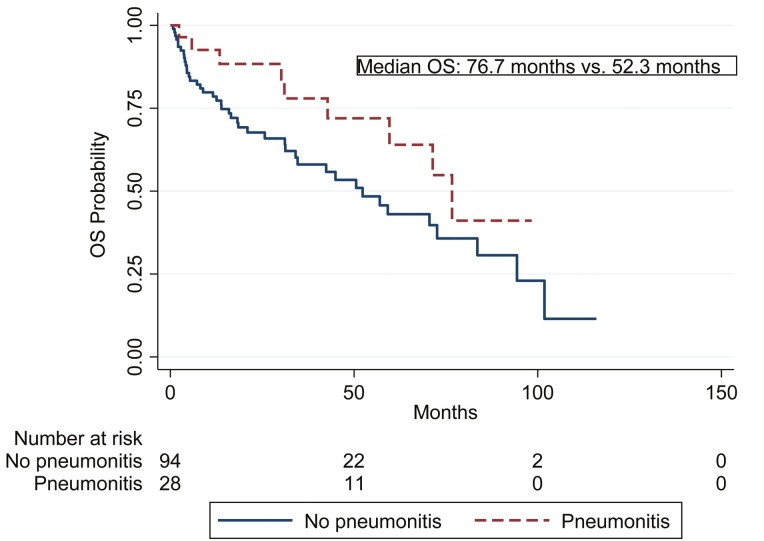
Association between everolimus-induced pneumonitis and Kaplan-Meier estimate for overall survival (OS).

At a median follow-up of 43.3 months (95% CI 30.6-60.0), 52 deaths (42.6%) were recorded with a median OS of 59.6 months (95% CI 42.8-83.5), from the start of everolimus. Patients treated with everolimus who developed pneumonitis had a numerically longer OS (76.7 months, 95% CI 42.8-not reached) than patients who did not develop pneumonitis of any grade (52.3 months, 95% CI 31.3-83.6; *P* = .093; Fig. 2); HR 0.54 (95% CI 0.26-1.12; *P* = .098). When the survival analysis was restricted to those patients who had at least 3 months of follow-up (landmark analysis; 8 patients with early death were excluded); the effect on survival remained numerically similar albeit without statistical significance [HR 0.55 (95% CI 0.26-1.21). *P* = .13].

In the multivariable Cox regression, higher age at the start of everolimus (HR 1.05, 95% CI 1.02-1.08; *P* < .001) and the use of everolimus in third line or higher setting (HR 2.73, 95% CI (1.42-5.23; *P* = .002) were associated with an increased risk of death, while the development of EiP of any grade (HR 0.39, 95% CI 0.19-0.82; *P* = .013) was shown to be an independent protective factor for death (Table 4).

**Table 4.** Univariable and multivariable Cox regression analysis for OS.

Variable	Univariable	*P*-value	Multivariable	
	HR (95% CI)		HR (95% CI)	*P*-value
Age at start of everolimus	1.03 (1.01-1.06)	.002	1.05 (1.02-1.08)	<.001
Presence of lung disease Yes No	1.09 (0.51-2.34) Reference	.807	—	—
COPD Yes No	0.80 (0.11-5.90) Reference	.834	—	—
Smoking history Yes No	0.85 (0.45-1.61) Reference	.628	—	—
Histological grade Grades 2 and 3 Grade 1	1.40 (0.76-2.57) Reference	.279	—	—
Primary Pancreatic Nonpancreatic	0.92 (0.52-1.61) Reference	.781	—	—
Lung metastases Yes No	0.85 (0.42-1.71) Reference	.669	—	—
Rash Yes No	0.71 (0.40-1.25) Reference	.243	—	—
Pneumonitis Yes No	0.54 (0.26-1.12) Reference	.098	0.39 (0.19-0.82)	.013
Line of treatment Third or further lines First or second lines	1.87 (1.03-3.44) Reference	.041	2.73 (1.42-5.23)	.002

COPD, chronic obstructive pulmonary disease; OS, overall survival.

**Figure 3.**
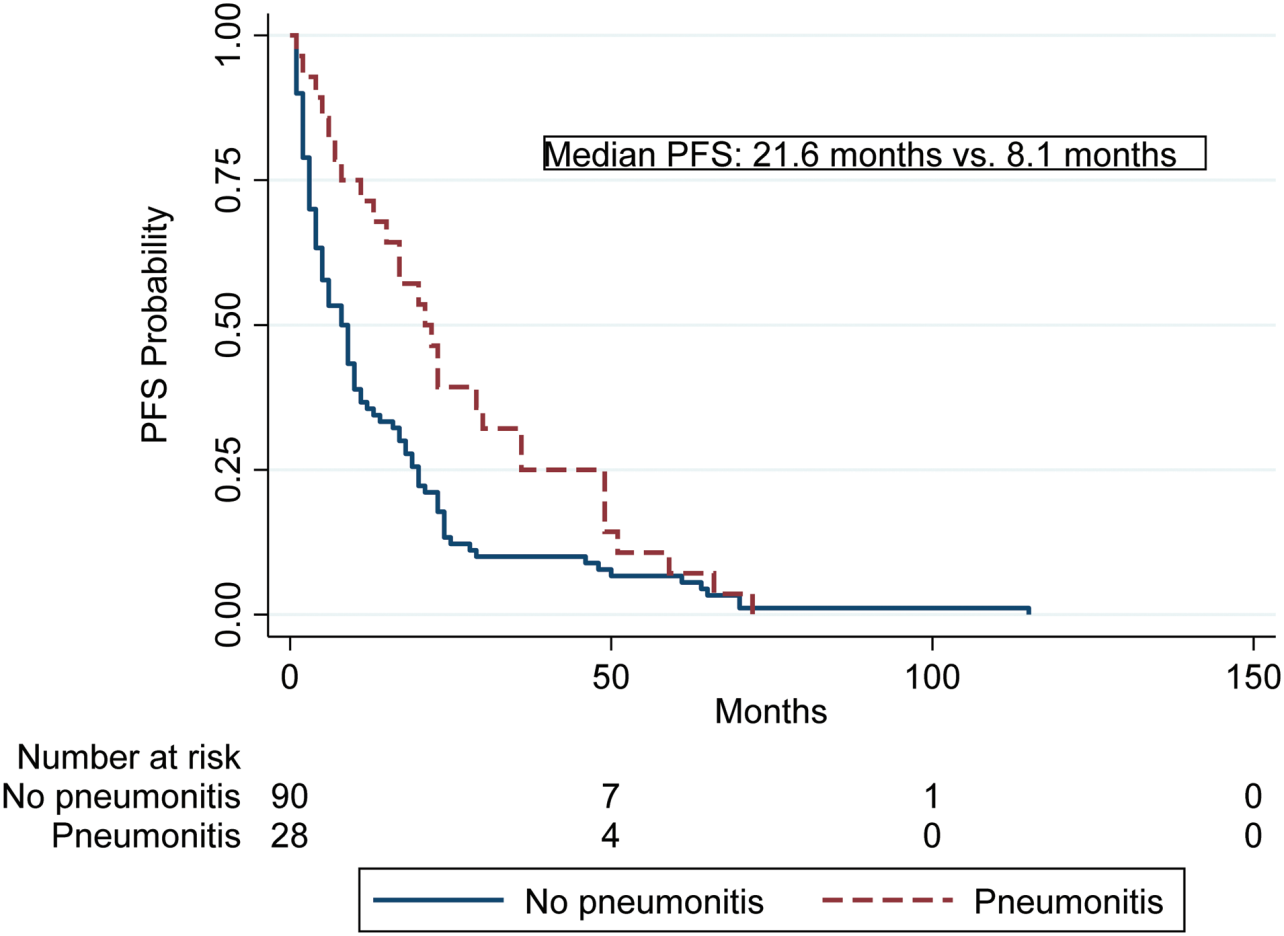
Association between everolimus-induced pneumonitis and Kaplan-Meier estimate for progression-free survival (PFS).

The PFS was significantly longer in the patients with EiP, 21.6 months (95% CI 13.4-31.0), versus 8.1 months (95% CI 5.1-10.4) in the patients without pneumonitis (HR 0.57, 95% CI 0.37-0.87; *P* = .010; Figure 3). Overall, the median time on everolimus was 5.5 months (0.23-117.2) in those that did not develop pneumonitis, and 17.9 months (0.83-74.0) if pneumonitis occurred (*P* = .0018). One patient was taking everolimus for almost 10 years (117 months) and was still on treatment at the data cut-off point.

## Discussion

In this study, the incidence of all-grade EiP in patients with NETs, 22.9%, was found to be higher when compared with the clinical trials reported rates, 12%-17%.^2-4^ Compared with real-world data, all grade pneumonitis and grades 3-4 (4%) was similar to an Italian retrospective study, showing an incidence of 18.9% (all grade) and 8.3% (grades 3-4) retrospectively^10^ and an American study which reported 21% all-grade pneumonitis.^14^ The lack of uniform diagnostic criteria may have contributed to the differences in the reported incidence.

Patients who developed EiP had numerically longer survival. Survival results did not change when conducting landmark analysis, adjusting the analysis to account for the fact that EiP is expected to be associated with time-on-treatment. Similarly, patients with pneumonitis had longer PFS and time on everolimus treatment. In metastatic RCC, mTOR-induced pneumonitis was associated with longer median PFS^15^ and median OS.^16, 17^ Recently, EiP was also associated with longer PFS and OS in patients with metastatic breast cancer in both unadjusted and adjusted analysis for prognostic factors, and also after performing a landmark analysis.^18^ In our cohort, the difference in OS was not statistically significant in the univariate analysis, albeit numerically longer for those with EiP, potentially because of the small number of patients treated, as NET is a rare disease, less aggressive (grade 1 or 2) and with several lines of therapies.

The present study did not identify any risk factors for EiP. This contrasts with a retrospective radiographic pattern-approach that identified a higher incidence of pneumonitis in never smokers^14^ and with a retrospective study which found that PRRT and chemotherapy increased risk for severe toxicity.^10^.

Everolimus-induced pneumonitis is a class effect AE, also seen with other mTOR inhibitors such as temsirolimus and sirolimus. The exact mechanism of development of mTOR inhibitor-related pneumonitis remains unknown. A study investigating sirolimus-associated pneumonitis reported the presence of lymphocytic alveolitis in bronchoalveolar lavage of all 8 patients analyzed, mostly of the CD4 type, suggesting a cell-mediated autoimmune response.^19^ While there are conflicting studies about the direct toxic effect and dose-related effect^19, 20^, there are an increasing number of preclinical studies suggesting that pulmonary inflammation could be due to cytokine production by mTOR inhibitors.^21^

As demonstrated in the current study and in others, EiP could be favorably associated with outcomes, but the potential underlying mechanism has not yet been elucidated. It could be hypothesized that an immunological effect impacts on its toxicity and the efficacy. The link between toxicity and efficacy was shown in studies evaluating molecular targeted therapies, as the development of rash on monoclonal antibodies targeting the epidermal growth factor receptor resulted in better outcomes in patients with colorectal and head and neck cancer.^22^

There is a lack of evidence-based management strategies on EiP, especially for grades 1-2. In this cohort, 8 of 9 patients that developed grade 1 pneumonitis had complete resolution with dose reduction or temporary interruption. This is in line with a management algorithm based in a comprehensive literature review.^23^ Management of the symptomatic patients should include a broad range of differential diagnoses, including infections that are also more frequent in patients using everolimus. A previous study by our group showed that 30.6% of patients on everolimus for NETs experienced infections of any grade; 21% had a serious infection and 7% had at least one opportunistic infection.^11^

Limitations of the current study were the retrospective design, without matched baseline characteristics in both groups, and that not all images were individually reviewed for pneumonitis (only the ones where the report had suspected findings). Of note, the 2 centers are specialized in the treatment of patients with NETs and have a multidisciplinary approach involving experienced radiologists.

The pandemic caused by the SARS-CoV-2 and the pulmonary imaging findings related to COVID-19 increase the difficulties in interpreting CT findings from patients receiving everolimus. Careful clinical evaluation is of paramount importance in this context. In this study, given its time span, COVID-19 was not a differential diagnosis.

## Conclusion

In conclusion, EiP of any grade can develop in nearly one quarter of patients with NETs, and it seems to be higher than previously reported in clinical trials. It often occurs in the first months of treatment with everolimus and is mostly uncomplicated. While no specific risk factors were identified in this study, patients developing EiP had longer PFS and a longer median OS. Further research is warranted to explore the association of EiP in patients with NETs with oncological outcomes.

## Data Availability

The data underlying this article will be shared on reasonable request to the corresponding author.
